# Editorial: Resistance of plants to parasitic nematodes and its application in breeding

**DOI:** 10.3389/fpls.2024.1439535

**Published:** 2024-06-06

**Authors:** Shaojie Han, Willibald Schliemann, Shiming Liu

**Affiliations:** ^1^ Key Laboratory of Biology of Crop Pathogens and Insects of Zhejiang Province, State Key Laboratory of Rice Biology and Breeding, Institute of Biotechnology, Department of Plant Protection, Zhejiang University, Hangzhou, Zhejiang, China; ^2^ Department of Cell and Metabolic Biology, Leibniz Institute of Plant Biochemistry, Halle (Saale), Germany; ^3^ State Key Laboratory for Biology of Plant Diseases and Insect Pests, Institute of Plant Protection, Chinese Academy of Agricultural Sciences, Beijing, China

**Keywords:** root-knot nematode, soybean cyst nematode, plant resistance, flavonoid biosynthesis, venom allergen-like proteins (DdVAP2)

The Research Topic “Resistance of Plants to Parasitic Nematodes and Its Application in Breeding” compiles six significant studies that advance our understanding of plant resistance to nematodes and their application in breeding programs. This editorial synthesizes these studies, emphasizing their collective contributions to sustainable agriculture through genetic and biotechnological innovations.

## Breeding for durable resistance

Huang et al. utilized CRISPR/Cas9 gene editing system to mutate the susceptibility gene *OsHPP04* in rice, leading to enhanced resistance to the rice root-knot nematode (*Meloidogyne graminicola*). The edited rice plants showed improved immune responses without any adverse agronomic traits, demonstrating CRISPR/Cas9 gene editing system’s potential in developing nematode-resistant crops and contributing to sustainable agriculture and crop protection. Devran et al. identified a new resistance gene, *RRKN1*, in the tomato line MT12, which is effective against root-knot nematodes (RKNs) even at high soil temperatures. Mapping *RRKN1* to chromosome 6 using Kompetitive Allele Specific PCR (KASP) markers offers a genetic resource for breeding tomato varieties with stable resistance under high-temperature conditions.

## Genetic insights into nematode resistance

Zhang et al. conducted an integrated set of transcriptome and metabolome analyses to reveal that flavonoids enhance rice resistance to *M. graminicola*. By comparing resistant (ZH11) and susceptible (IR64) rice varieties, the study highlighted increased expression of genes and metabolites involved in flavonoid biosynthesis and cell wall construction in the resistant variety, providing insights for developing nematode-resistant rice varieties. Lian et al. investigated 12 soybean cultivars for resistance to soybean cyst nematode (SCN) *Heterodera glycines*. By evaluating marker haplotypes at the *rhg1* and *Rhg4* loci and testing resistance against multiple SCN races, all cultivars were found to exhibit Peking-type resistance, effective against multiple SCN races. These findings offer valuable genetic resources for breeding SCN-resistant soybeans. Mahmood et al. performed genome-wide association studies (GWAS) to identify quantitative trait nucleotides (QTNs) associated with SCN resistance in soybean breeding lines. The study identified multiple resistance loci and candidate genes, providing valuable insights for breeding SCN-resistant soybeans and diversifying resistance sources in North American breeding programs.

## Role of venom allergen-like proteins

Chang et al. studied the role of DdVAP2 in the parasitism of *Ditylenchus destructor*, a nematode affecting potatoes and sweet potatoes. The research identified DdVAP2’s expression in nematodes and its secretion into host plants. RNAi experiments showed that knock-down of DdVAP2 reduced nematode infection and reproduction, highlighting its crucial role in parasitism.

## Integrating findings for sustainable agriculture

Collectively, these studies underscore the critical role of genetic and biotechnological approaches in developing nematode-resistant crops. By enhancing our understanding of the molecular mechanisms of resistance and identifying new resistance genes and loci, these findings pave the way for innovative breeding strategies. The integration of these advancements into breeding programs holds promise for sustainable agricultural practices, reduced reliance on chemical nematicides, and improved crop yields and food security.

## Author contributions

SH: Writing – original draft, Writing – review & editing. WS: Writing – review & editing. SL: Writing – original draft, Writing – review & editing.

